# Outcomes of Chronic Hemodialysis Patients in the Intensive Care Unit

**DOI:** 10.1155/2013/715807

**Published:** 2013-05-09

**Authors:** Melanie Chan, Marlies Ostermann

**Affiliations:** Department of Critical Care, King's College London, King's Health Partners, Guy's & St Thomas' Foundation Hospital, London SE1 7EH, UK

## Abstract

Patients with end-stage renal disease (ESRD) experience higher rates of hospitalisation, cardiovascular events, and all-cause mortality and are more likely to require admission to the intensive care unit (ICU) than patients with normal renal function. Sepsis and cardiovascular diseases are the most common reasons for ICU admission. ICU mortality rates in patients requiring chronic hemodialysis are significantly higher than for patients without ESRD; however, dialysis patients have a better ICU outcome than those with acute kidney injury (AKI) requiring renal replacement therapy suggesting that factors other than loss of renal function contribute to their prognosis. Current evidence suggests, the longer-term outcomes after discharge from ICU may be favourable and that long-term dependence on dialysis should not prejudice against prompt referral or admission to ICU.

## 1. Introduction

The incidence and prevalence of end-stage renal disease (ESRD) is rising worldwide, in part, due to the increasing rates of diabetes, hypertension, and an ageing population [1, 2]. ESRD patients experience higher rates of hospitalisation, cardiovascular events, and all-cause mortality when compared to patients with normal renal function, and are more likely to require admission to the intensive care unit (ICU) [3, 4]. It is estimated that 2% of chronic dialysis patients require admission to ICU every year [5]. The presence of established end-stage organ failure and often numerous comorbidities can impact on decisions regarding escalation of care and ICU admission. It was previously assumed that patients requiring long-term dialysis would have similar ICU outcomes to those with acute kidney injury (AKI) requiring acute RRT; however, emerging evidence suggests otherwise. 

## 2. Admission Rates to ICU 

Chronic dialysis patients have significantly higher critical care admission rates than the general population, with a calculated 15.6 admissions per 100 prevalent patients with ESRD per year versus 0.58 per 100 prevalent patients without ESRD per year [6]. The largest cohort of critically ill ESRD patients studied derives from the Intensive Care National Audit & Research Centre (ICNARC) Case Mix Programme Database which records patient data from more than 200 ICUs across the UK. Analysis of this database showed that from 1995 to 2004, there were 270,972 admissions to ICU, of whom 1.3% were chronic dialysis patients [4]. This was estimated to be equivalent to six ICU admissions or 32 ICU bed days per 100 dialysis patient-years. When compared to annual ICU admission rates of 2 per 1,000 of the general population, this represents a 30-fold difference in critical care requirements. 

Other studies have proposed higher admission rates from 3.4% to 8.6% [6–8]; however, these are mostly single centre studies and involve smaller study cohorts than ICNARC [4]. Although ICU admission rates for the ESRD population vary from 1.3% to 8.6%, it is evident that chronic dialysis patients place a larger burden on critical care resources than the general population. 

## 3. Characteristics of Critically Ill Dialysis Patients

### 3.1. Reasons for Admission to ICU

Data from the ICNARC cohort show that dialysis patients are more likely to be admitted to ICU with a medical diagnosis than those without ESRD (66.7% versus 56.2%) [4] and are less frequently admitted to ICU after elective surgery (7.4% versus 19%, *P* < 0.0001). Admission rates after emergency surgery are comparable between those with and without ESRD [6].

Cardiovascular disease and sepsis constitute two of the most common reasons for admission to ICU [1, 2]. Dialysis patients are particularly susceptible to infections due to uraemia-related immune deficiency, defective phagocytic function, older age, and comorbidities including diabetes mellitus. In addition, repeated vascular access for the purpose of hemodialysis increases the risk of bacteraemia. Annual percentage mortality rates related to sepsis for dialysis patients have been estimated at 100- to 300-fold higher than the general population [9]. Between 5.6% to 46% of chronic dialysis patients are admitted to ICU with a diagnosis of sepsis [4, 6, 7, 10–15]. Significantly, more ESRD patients are admitted to ICU with sepsis compared to those without renal failure (15.8% versus 6.5%, *P* < 0.0001) [6]. A small Brazilian study reported that the lung was the most frequent source of sepsis, followed by soft tissue, catheter-related/bloodstream, and abdominal sources [14]. 

Patients with renal dysfunction have a higher risk of adverse cardiac events including myocardial ischemia, pulmonary oedema, cardiogenic shock, arrhythmias, and sudden cardiac death. Studies have estimated that the proportion of ESRD patients admitted to ICU with a cardiac diagnosis (including pulmonary oedema) ranges from 5.1% to 31% [4, 6, 7, 10–14]. Hemodialysis patients have a 10-fold increased risk of dying from cardiac arrest than the general population [2]. ICU admission after cardiopulmonary resuscitation (CPR) is more common in ESRD patients (13.6% versus 7.3%, *P* < 0.001) [4], and the fluid and electrolyte shifts experienced during and in between dialysis sessions may contribute to this increased risk. Other predisposing factors are left ventricular hypertrophy/dysfunction, ischemic heart disease, autonomic dysfunction, hypertension, diabetes, and being male [16]. 

Gastrointestinal bleeding is the third most common reason for chronic dialysis patients to require critical care, with studies estimating rates of 2.7% to 20% in critically unwell dialysis patients [7, 10, 13, 14]. 

It is difficult to ascertain precisely how often ESRD patients require critical care intervention for complications directly related to renal failure and/or dialysis, including pulmonary oedema, arrhythmias, hyperkalaemia, or vascular access-related septicaemia. Hutchison and colleagues [4] report that the most common ICU admission diagnosis for long-term dialysis patients is “chronic renal failure” (8.6%), which they define as volume overload or electrolyte disturbance. Hyperkalaemia was recorded as the admitting diagnosis for 4.3% [7] and 3% [11] of ESRD patients admitted to single ICUs in France and Australia, respectively. Clearly, these statistics depend on not only patients' severity of illness and long-term prognosis but also ICU admission policy and capacity, patients' wishes, and whether there is a renal unit in the hospital or not. 

### 3.2. Severity of Illness Scores

Chronic dialysis patients requiring ICU care are more critically unwell and have a greater number of comorbidities than the general population. Strijack et al. [6] found that they had significantly higher rates of diabetes (52.3% versus 21.7%, *P* < 0.0001) and peripheral arterial disease (29.7% versus 12.3%, *P* < 0.0001) than those without ESRD on admission to the ICU. Rates of coronary artery disease, stroke, and cancer were comparable between the two groups. 

Several studies have used ICU mortality and prognostication models such as the Acute Physiology and Chronic Health Evaluation (APACHE) II score [17] to attempt to quantify the severity of illness of dialysis patients admitted to critical care. Hutchison et al. [4] reported that both the APACHE II (24.7 versus 16.6, *P* < 0.001) and Simplified Acute Physiology Score (SAPS) (17.2 versus 12.6, *P* < 0.001) were significantly higher in dialysis patients when compared to those without chronic renal failure. A similar trend was seen in a Canadian historical cohort study where patients with ESRD had a higher APACHE II score than those without ESRD (24 versus 15, *P* < 0.0001), a finding that persisted even after removal of the renal component (serum creatinine and the presence of AKI) of the score (20 versus 14, *P* < 0.0001) [6]. 

The reasons why ESRD patients are more critically unwell on admission to ICU than the general population are not fully understood. It may reflect the differing admission diagnoses between the groups. It also raises the possibility that there exists a higher threshold for seeking intensive care intervention in chronic dialysis patients, resulting in delayed referral. Another potential explanation may be that chronic dialysis patients need to be more critically unwell before being accepted into the ICU. 

In summary, patients admitted to critical care on long-term dialysis are more likely to have multiple comorbidities and have a higher severity illness score on admission than the general population. They more frequently present having had a cardiac arrest and CPR prior to admission and are more commonly admitted for medical rather than surgical reasons [15].

## 4. Short-Term Outcomes

### 4.1. Mortality

During the last ten years, numerous studies have focussed on the outcomes of critically ill long-term dialysis patients admitted to ICU (Table 1). Prior to this, it was assumed that ICU mortality in this population was comparable to that of patients with AKI. Reliable data on prognosis are necessary to enable ESRD patients and their physicians to make well-informed and timely decisions regarding escalation of care.

Clermont et al. [8] reported an observed ICU mortality of 11% for ESRD patients compared to 5% in patients without renal failure. Other studies have reported ICU mortality rates of 9% to 44% for chronic dialysis patients [4, 5, 7, 8, 10–15, 18–20]. 

Analysis of the UK ICNARC database showed an ICU mortality rate of 26.3% in patients with ESRD compared to 20.8% in those without ESRD (*P* < 0.001) [4]. This significant increase in mortality is however not surprising, given the higher illness severity scores of ESRD patients on admission to ICU in this study. In 199 dialysis-dependent patients requiring support of two or more organ systems (including RRT), ICU mortality was 44% [18], a figure similar to ICU mortality for patients with multiorgan failure which can range from 20% to 95% depending on the number of organs involved and underlying comorbidity. 

Factors associated with ICU mortality in chronic dialysis patients are age, number of nonrenal organ system failures, an abnormal serum phosphorus level (high or low), higher mean APACHE II or SAPS II score, and duration of mechanical ventilation [7, 8, 11]. There is clearly some overlap between these factors as confirmed by multivariate analyses [7]. The importance of abnormal serum phosphorus levels is unclear. Manhes et al. [7] hypothesise that a low phosphate level can signify malnutrition and be related to severity of illness, whereas hyperphosphataemia may be an indicator of inadequate renal replacement and a risk factor for cardiovascular disease.

### 4.2. Length of Stay

Chronic dialysis patients have comparable lengths of stay in ICU to the general population [4, 6–8], with mean length of stay ranging from 1.9 to 9 days [4, 6–8, 11, 14, 18, 21, 22]. Some of the discrepancies between different studies may be due to differences in discharge policies and level of staffing on the receiving ward. In hospitals with renal units offering level two care, safe discharge of patients may be possible earlier compared to hospitals without dedicated step-down units. 

### 4.3. Readmission to ICU

ESRD patients have a higher rate of readmission to ICU during the same hospital stay than patients with normal renal function [4, 6], with quoted figures of 9% to 12% [4, 6, 11]. Strijack et al. [6] found a significant difference in readmission rates (12% versus 4.9%, *P* < 0.0001) between those on chronic dialysis and the general population and reported twice the frequency of readmissions to ICU within three days in the former. Dialysis modality and vascular access had a significant impact on ICU readmission rates; hemodialysis patients using arteriovenous (AV) fistulae as opposed to central venous catheters (CVCs) had significantly reduced readmissions (4.7% versus 16.4%, *P* < 0.05) [15], but it was acknowledged that CVCs may be a surrogate for poor performance status. Dialysis dependence was also independently associated with two-fold higher odds for ICU readmission in the elderly (>65 years) population even after adjustment for case mix and severity of illness variables [23].

Evidence suggests that chronic dialysis patients have comparable stays in ICU to those without ESRD but experience almost twice the number of readmissions. Readmission to ICU is associated with poor outcomes, and while many renal units have considerable experience in managing unwell dialysis patients, careful planning for a timely and safe discharge from ICU to a suitable destination is paramount. 

## 5. Longer-Term Outcomes

Hospital, 30-day, and 90-day mortality rates for critically ill chronic dialysis patients are estimated at 14% to 56% [4–8, 10, 11, 13, 14, 18, 21, 22], 32% to 41% [10, 13, 22], and 42% to 44.6% [12, 24], respectively. Hospital mortality rates were significantly higher in chronic dialysis patients compared to the non-ESRD population after ICU discharge (45.3% versus 31.2%, *P* < 0.001) [4]. 6-month and 12-month mortality rates for critically ill dialysis patients have been reported as 38% and 48%, respectively [7, 15, 20]. 

In one study, the long-term mortality in critically unwell ESRD patients was 25 times higher than expected from mortality rates in the general population (standardized mortality ratio 25; 95% confidence interval 20–31), with the highest number of deaths occurring in the first year after ICU discharge [12]. In contrast to this, Chapman et al. [18] reported that the majority of deaths occurred within the first month of ICU admission. Hemodialysis patients, who survived to one month or hospital discharge, had long-term survival rates equivalent to ESRD patients who had not been admitted to ICU. Bagshaw et al. [20] found that chronic dialysis patients had a similar 1-year mortality rate to those with no kidney dysfunction after adjustment for age, severity of illness, and admission type, a finding confirmed by Strijack et al. [6]. This suggests that although ESRD identifies a cohort with a worse ICU outcome than the general population, the prognosis is related to illness severity and comorbidities rather than the lack of renal function itself.

Medical diagnoses, diabetes and heart failure are all significant predictors of longer-term mortality in chronic dialysis patients admitted to ICU [4, 12, 18]. Age, dialysis vintage and APACHE II score did not correlate with mortality [18]. Hemodialysis patients with CVC access had higher crude mortality rates at both 6 and 12 months than those with AV fistulae [15]. CVCs remained independently associated with death even after adjustment for baseline and ICU admission characteristics as well as comorbidities. Again, this finding is open to confounding, given that tunnelled lines are more commonly used in patients with a poor performance status or limited life expectancy, and pose an increased risk of infection. 

Older age, admission after emergency surgery, chronic health problems, CPR in the 24 hours preceding admission to ICU, having been in hospital for at least 7 days prior to ICU, and the number of failed nonrenal organ systems significantly affect outcomes of ESRD patients after ICU [4, 8, 10, 14]. As expected, physiological, and biochemical disturbances including hypotension, bradycardia, tachypnoea, hypoxia, reduced Glasgow Coma Score, hyponatraemia, leucopenia, and sepsis within the first 24 hours of ICU admission exert a significant impact on hospital mortality, too [4]. Mechanical ventilation and the need for inotropic support are also significantly associated with mortality at 30 days [13]. Whilst many of these variables are risk factors for mortality in ICU patients in general, their impact on the ESRD population appears to be greater, perhaps due to a lack of physiological reserve in this group. 

## 6. ICU Outcomes in AKI Compared to ESRD 

AKI is common in critically ill patients and a frequent reason for admission to ICU. A significant proportion require RRT, and has a high-associated mortality rate which can vary from 25% to 90% depending on the patient's characteristics and associated organ dysfunction.

Clermont and colleagues [8] were among the first to examine ICU mortality in patients with AKI, ESRD, and those with normal renal function. In spite of similar illness severity scores in the AKI and ESRD populations, ICU mortality rates were five times higher in AKI patients who required RRT than those on chronic dialysis and ten times higher when compared to those with normal renal function (57% versus 11% versus, 5%, resp.). Similarly, in another study, ICU and hospital mortality rates were twice as high in AKI patients requiring RRT compared to ESRD patients when matched for age, severity of illness, and number of organ dysfunctions (42% versus 20% and 50% versus 24%, resp.) [14]. 

A large retrospective database analysis found similar results when comparing the outcomes of 1847 critically ill patients with AKI on RRT to 797 ESRD patients admitted to ICU [19]. ESRD patients had approximately half the ICU and hospital mortality rates of AKI patients on RRT (20.8% versus 54.1%, *P* < 0.0001 and 34.5% versus 61.6%, *P* < 0.0001, resp.). As expected, increasing ICU mortality was seen with an increasing number of organ failures in both cohorts; however the group of AKI patients on RRT had a significantly higher proportion with more than two nonrenal organ failures (75.4% versus 25.6%). Length of stay in both ICU and hospital was also significantly increased in patients with AKI compared to ESRD [14, 19, 22].

Although the majority of the published literature indicates that ICU and hospital outcomes are significantly worse for AKI patients requiring RRT than critically ill chronic dialysis patients, two small studies have reported comparable ICU and hospital/90-day mortality rates for diagnosis and severity-score matched AKI and ESRD patients receiving continuous RRT [5, 24]. 

Patients with AKI were more likely to require mechanical ventilation and vasopressors than those on chronic dialysis, even when matched for severity of illness and controlled for mode of RRT [14, 19, 22], a finding which may explain their increased mortality rate. The strongest independent risk factors for ICU mortality in both cohorts were mechanical ventilation, maximum number of organ failures, and nonsurgical reason for admission [19, 22]. 

## 7. Validity of ICU Severity Scores in ESRD

ICU illness severity and organ dysfunction scoring systems, including APACHE II and III [17, 25], SAPS II [26] and sequential organ failure assessment (SOFA) [27] scores, are primarily used within critical care as research and audit tools to enable comparison between observed and predicted mortality and controlled matching between study cohorts. Whilst these scoring systems have been validated in a wide variety of different subspecialties, their application to ESRD patients remains controversial. 

Hutchison and colleagues [4] evaluated the APACHE II score and reported an area under the receiver operating curve (AUC) of 0.721 for their ESRD cohort, compared to 0.805 in the non-ESRD group, indicating that it is less accurate in predicting mortality in chronic dialysis patients. When using a modified renal-adjusted APACHE II score especially for dialysis patients the AUC improved to 0.817. Uchino et al. [5] and Juneja et al. [13] also reported an AUC of 0.81 and 0.86, respectively, for the APACHE II score, using a much smaller cohort of long-term dialysis patients. The APACHE III score has been found to overestimate 30-day mortality in ESRD [8, 10]. Similarly, Strijack et al. [6] found that the APACHE II score over predicted mortality in dialysis patients by a factor of 2.5. 

Data on the validity of the SOFA score in patients with ESRD are conflicting. One study reported an AUC of 0.92 (although not significantly different from the APACHE II score) [13], whereas Dara et al. [10] found the SOFA score to be less accurate than the APACHE III score with an AUC of 0.66. Notably, the patients in the first study were sicker than those included in the latter with a higher number of organ failures and greater need for mechanical ventilation and inotropes. 

The Stuivenberg Hospital Acute Renal Failure Scoring System (SHARF II) is a renal specific prognostic score designed to predict outcomes in patients with AKI [28]. Based on eight parameters (age, serum albumin, bilirubin, prothrombin time, respiratory support, sepsis, hypotension and heart failure), two scores are calculated at AKI diagnosis and 48 hours later. In a cohort of 293 patients admitted to the ICU with AKI, AUC was 0.82 at diagnosis and 0.83 at 48 hours. Validation of this score in critically ill long-term dialysis patients is awaited. 

At present, there is limited and conflicting information regarding the validity of commonly used scoring systems in chronic dialysis patients. The majority of studies have used too small sample sizes to make any reliable claims. As mentioned previously, ESRD patients have similar illness severity scores to patients with AKI on admission to ICU but have significantly better outcomes indicating that these prognostic tools overestimate mortality in dialysis patients. The application of these tools in their current form to a population of anuric patients with chronically deranged biochemistry on long-term RRT is at best limited. 

## 8. Conclusion

Critically ill patients with ESRD are frequently admitted to ICU, and although they display worse outcomes than those with normal renal function, their prognosis is better than that of patients with AKI requiring RRT. Mortality is related primarily to the severity of the underlying illness and their comorbidities rather than to lack of renal function itself. Having survived an episode of critical illness, data on longer-term outcomes remains conflicting, and little is currently known about quality of life and performance status after discharge from ICU. Prognostic scoring systems used in critical care appear to overestimate mortality in the chronic dialysis population and should be used with caution. Current evidence suggests that long-term dependence on dialysis should not prejudice against prompt referral or admission to ICU.

## Figures and Tables

**Table 1:** Comparison of studies assessing outcomes of chronic dialysis patients admitted to ICU.

Study	Country	Year of publication	No. of patients (*n*)	Mean age (years)	Mean severity score on admission to ICU	ICU mortality (%)	Hospital mortality (%)	30-day mortality (%)	ICU LOS (days) mean ± SD or median [range]	ICU readmission rate (%)
Clermont et al. [8]	USA	2002	57	58	64 (APACHE III)	11	14	—		—
Uchino et al. [5]	Australia	2003	38	45	22 (APACHE II)	22	38	—	6	—
Dara et al. [10]	USA	2004	93	66	64 (APACHE III)	9	16	22	2	—
Manhes et al. [7]	France	2005	92	63	49.4 (SAPS II)	28	38	—	6.2 ± 9.9	—
Bagshaw et al. [20]	Canada	2006	92	66	29.7 (APACHE II)	16	34	—	—	—
Hutchison et al. [4]	UK	2007	3420	57	24.7 (APACHE II)	26	45	—	1.9 [0.9–4.2]	9
Ostermann and Chang [19]	UK/Germany	2008	797	55	8 (SOFA)	21	35	—	2 [1–64]	—
Senthuran et al. [11]	Australia	2008	70	57	26.1 (APACHE II)	17	29	—	2 [1–27]	—
Strijack et al. [6]	Canada	2009	619	62	24 (APACHE II)	—	16	—	4.3	12
Chapman et al. [18]	UK	2009	199	59	27.6 (APACHE II)	44	56	—	7.5 ± 10.1	—
Rocha et al. [14]	Brazil	2009	54	66	43.9 (SAPS II)	20	24	—	5 [3–11]	—
Juneja et al. [13]	India	2010	73	54	27.1 (APACHE II)	27	—	41	2 [1–20]	—
Sood et al. [15]	Canada	2011	578	61	19 (APACHE II, renal adjusted)	13	—	—	—	—
Walcher et al. [22]	USA	2011	28	58	—	36	39	39	9 ± 8	—
O'Brien et al. [21]	UK	2012	8991	59	24.6 (APACHE II)	24	42	—	2 [0.9–4.7]	—

Abbreviations: APACHE: Acute Physiology Assessment and Chronic Health Evaluation; SOFA: sequential organ failure assessment; SAPS: Simplified Acute Physiology Score; ICU: intensive care unit; LOS: length of stay.
